# Highly Efficient Use of Infrared Spectroscopy (ATR-FTIR) to Identify Aphid Species

**DOI:** 10.3390/biology11081232

**Published:** 2022-08-18

**Authors:** Roma Durak, Beata Ciak, Tomasz Durak

**Affiliations:** Institute of Biology and Biotechnology, University of Rzeszów, Pigonia 1, 35-310 Rzeszów, Poland

**Keywords:** entomology, chemotaxonomy, rapid species identification, mid-infrared spectroscopy

## Abstract

**Simple Summary:**

To date, the identification of aphids, which are serious pests for many plant species, is mainly based on their morphological features. The spectroscopic method, using the chemical composition of the aphid body, has been tested as an option for identifying species. Nine absorption peaks have been established to enable the accurate identification of 12 aphid species.

**Abstract:**

Aphids are commonly considered to be serious pests for trees, herbaceous and cultivated plants. Recognition and identification of individual species is very difficult and is based mainly on morphological features. The aims of the study were to suggest the possibility of identifying aphids through the use of Fourier-transform infrared (FTIR) spectroscopy, and to determine which absorption peaks are the most useful to separate aphid species. Using FTIR spectroscopy, based on the chemical composition of the body, we were able to distinguish 12 species of aphid. We have shown that using nine distinct peaks corresponding to the molecular vibrations from carbohydrates, lipids, amides I and II, it is possible to accurately identify aphid species with an efficiency of 98%.

## 1. Introduction

Entomological materials are used in many areas of research, from insect monitoring or epidemiology through to forensics. Therefore, new methods of precise species identification or determining the age of developmental stages are being sought. The method of spectroscopy, which ensures objective and quick identification of an insect, is increasingly becoming the norm. The premise that every species has a unique chemical composition allows the advantageous use of FTIR spectroscopy for taxonomic purposes. Near-infrared spectroscopy (NIRS) was used to quickly identify the age of the *Aedes albopictus* mosquito population in order to avoid a potential epidemic of, e.g., diseases such as dengue and Zika [1]. An attenuated total reflection–Fourier-transform infrared (ATR-FTIR) spectrometer for acquiring high-resolution mid-infrared (MIR) spectra (4000 cm^−1^ to 400 cm^−1^) was used to discriminate blood samples originating from a common vertebrate host, collected from the malaria vector, *Anopheles arabiensis*, and this method avoided the required molecular techniques [2,3]. The biochemical specificity of FTIR imaging results from the interaction of infrared light with the molecular vibrational modes of the molecules being interrogated and, thus, provides information about the biochemical composition. FTIR spectroscopy determining absorption spectra in the mid-infrared frequency region, which covers spectral regions that comprise a biochemical fingerprint, is important for interrogating biological materials [4,5]. Spectroscopic methods were used to detect flunitrazepam in larvae, puparia and adults of necrophagous flesh flies (*Chrysomya megacephala, Chrysomya albiceps* and *Cochliomyia macellaria*) [6,7]. Spectroscopic methods were used for a wide range of insect taxonomy problems; for example, within the Diptera order, they were used to discriminate individuals of *Drosophila subobscura* and *D. obscura* [8], and identify species within the Sarcophagidae family [9], to determine gender in fly pupae [10], identify stored grain beetles [11] and differentiate species of Lepidoptera [12,13]. Imaging spectroscopy has been used to distinguish different species and castes of ant and even two cryptic-species of ants [14,15,16]. Spectroscopic techniques were used to investigate the hidden lifestyle of arthropod pests [17]. They can provide data at the species level, and may also be useful in identifying local populations [18], subspecies [19], intraspecific age-classes [20], sexual dimorphism [10] and phylogeny [21]. ATR-FTIR spectroscopy is also widely used in forensic entomology. Accurate identification of necrophagous insect species is very important in determining the time of death and is a major obstacle for strengthening forensic entomology worldwide. The taxon that has been frequently found on cadavers resulting from homicides and natural death is Diptera [22]. Although spectroscopic methods have been used by taxonomists, to date, they have only been used among Hemiptera to identify a few species of leafhoppers [23,24].

Aphids are small sap-sucking insects and members of the superfamily Aphidoidea, which includes about 5000 species. These insects are considered taxonomically difficult due to their specific features. Due to their polymorphism, which means several kinds of morphs of adults in the same species, distinguishing between species is complicated. Most species produce 5 different morphs, but some can have 10, including stem mother, apterous viviparous parthenogenetic female, alate viviparous parthenogenetic female and oviparous female and male. Aphids are also characterized by complex life cycles, often associated with the change of host plants, and a holocyclic life cycle in which parthenogenetic and sexual generations alternate. Some aphids are monophages, but there are also oligophages and polyphages that can feed on many different plants. Aphids are considered to be serious pests for both trees and herbaceous plants, as well as cultivated plants. Recognition and discrimination of individual species is very difficult and is based mainly on morphological features. Therefore, it is important to search for, and use, new methods to identify these insects. The aims of the study were to (1) indicate the possibility of identification of aphids with the use of FTIR spectroscopy, and (2) determine which absorption peaks can be used to separate aphid species.

## 2. Materials and Methods

### 2.1. Sample Collection and Measurement

In total, 12 species of aphids were used in the research: *Acyrthosiphon pisum, Aphis fabae, Aphis pomi, Aphis viburni, Aphis solanella, Aphis spiraecola, Brachycorynella asparagi, Cinara cupressi, Cinara tujafilina, Macrosiphum rosae, Macrosiphonella artemisiae,* and *Uroleucon obscurum.* For each species, 5 individuals from different colonies were collected. The samples were collected in June (summer generation). Only the wingless adult females were analyzed. The material used for analyses included the entire body of fresh aphid individuals.

Individual aphids were placed in separate test tubes. Each aphid sample was smeared on a glass slide and a small amount of the matter was applied to cover the ATR-FTIR crystal [25]. Measurements were taken on aphid samples covered with a slide window and clamped to the ATR diamond crystal using pressure gauges. Spectra of aphid samples were measured using a Nicolet iN10 MX microspectrometer, which has a deuterated triglycine sulphate (DTGS) detector and KBr beam splitter (Thermo Fisher Scientific, Waltham, MA, USA) with Nicolet iZ10 module, coupled with a Smart Orbit ATR one-bounce diamond crystal accessory. Measurements were controlled using OMNIC software (Thermo Fisher Scientific). Spectra for both background and aphid sample measurements were recorded at a resolution of 4 cm^−1^ and 32 scans in the range of 4000–500 cm^−1^. For each spectrum, baseline correction and vector normalization were made using OPUS 7.0 software (Bruker Optik GmbH, Ettlingen, Germany).

### 2.2. Statistical Analyses

Statistical analyses were performed in two steps. In the first step, based on an entire spectral dataset, a search for the ATR-FTIR ranges of potential importance for distinguishing aphid species by Principal Component Analysis (PCA) was performed. PCA allows spectral dataset interpretation, taking into account the variability existing in the dataset. PCA gives hypothetical variables accounting for the variance in the dataset of original variables. To recognize the degree to which original variables enter into the hypothetical variables found by PCA, inspection of the loadings plots obtained based on PC coefficients was made. Loadings describe the relationships between original and hypothetical variables. Based on these loadings, the meaning of the variables was interpreted, and identification of the ATR-FTIR ranges that included the most important ones from the species differentiation point of view was performed. In the second step, based on the entire spectral dataset, the selected ATR-FTIR ranges were used to test their efficiency in differentiating aphid species. To do this, the visualization and estimation of the relationships between aphid species and maximum absorbance recorded in each of the selected ATR-FTIR ranges by discriminant analysis (LDA analysis), with group assignment by a leave-one-out cross-validation (jackknifing) procedure, were tested. To find the best result of species differentiation, we examined how the variation explained by aphid species was dependent on the ATR-FTIR ranges (expressed by maximum absorbance) selected for analysis, for every possible combination of ATR-FTIR ranges. As a result of selection, the ATR-FTIR ranges that gave the best LDA results were distinguished as a key ATR-FTIR range set, critical for the explanation of species differentiation. The relationship of the main axes of LDA analysis with selected key ATR-FTIR ranges was confirmed by Spearman’s rank correlation. Statistical analyses were performed using PAST software package 4.0 [26]. PCA was carried out on the variance–covariance matrix, with the singular value decomposition (SVD) algorithm. Before LDA analysis, to improve the distribution and interpretability of spectral dataset, data were square-root transformed and standardized. The LDA analysis classified the spectral data, assigning each point to the group that gave the minimum Mahalanobis distance to the group mean. The Mahalanobis distance was calculated from the pooled within-group covariance matrix (for computational details, please see reference manual for the PAST software in the link to the website) [27].

## 3. Results

### 3.1. Distinction of ATR-FTIR Ranges

**Figure 1:**
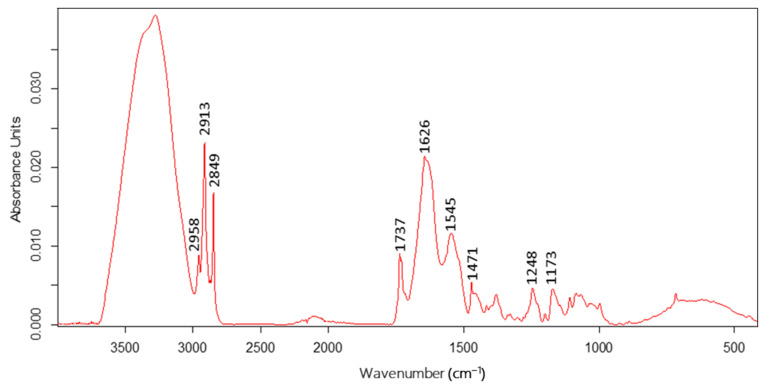
Averaged ATR-FTIR spectrum of aphid species.

The compared spectra of individuals from each species differed in the absorbance strength and peaks position, which is related to changes in the chemical composition of the insects’ bodies. The averaged ATR-FTIR spectrum of aphid species, with absorption peaks selected during the statistical analyses, is shown in Figure 1.

**Figure 2:**
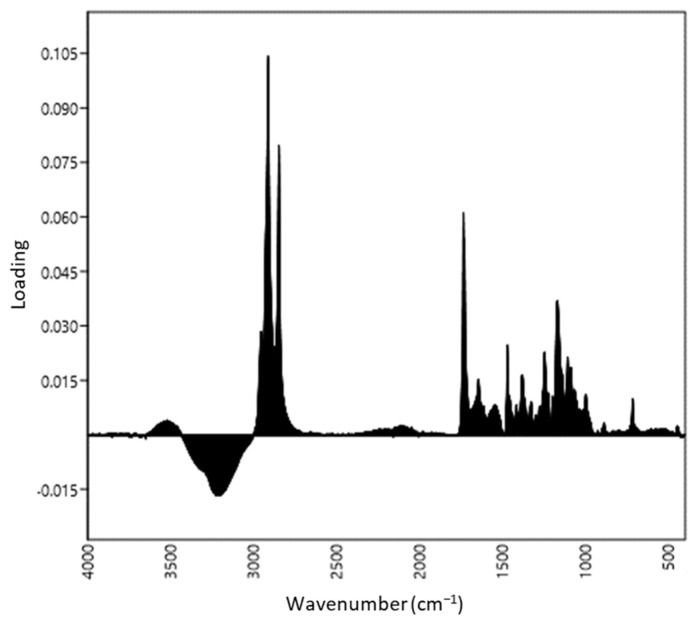
Loadings plots produced by PC 1 with peaks at 1173, 1248, 1471, 1737, 2849, 2913 and 2958 cm^−1^.

**Figure 3:**
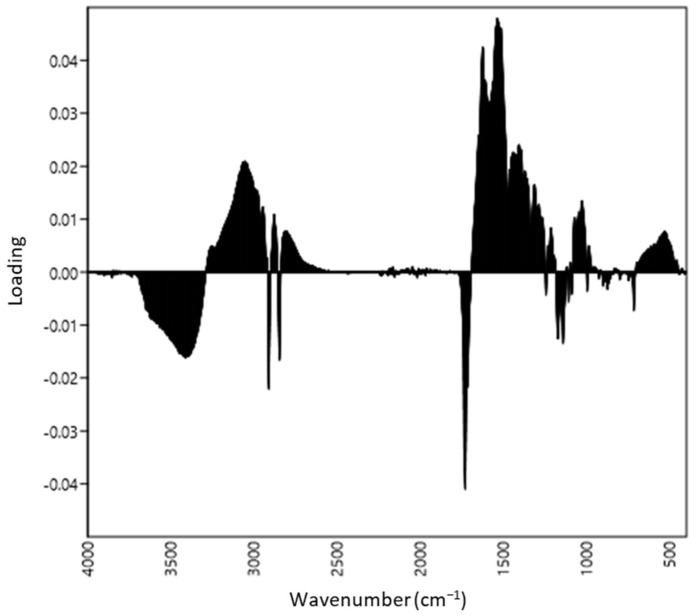
Loadings plots produced by PC 2 with peaks at 1408, 1545, 1626 and 1737 cm^−1^.

The first two PCA axes explained 76.7% and 16.1% of variance in the spectral dataset, respectively. The loadings plots presented in Figure 2 suggest that the differentiation between aphid species along the first PCA axis was mainly related to the molecular vibrations at 2958, 2913, 2849, 1737, 1471, 1248 and 1173 cm^−1^. In the case of the second PCA axis, the most decisive were 1737, 1626, 1545 and 1408 cm^−1^ (Figure 3). Based on these results, the important ATR-FTIR ranges from the sample separation point of view were determined.

### 3.2. Species Identification on the Basis of Chemical Composition

**Figure 4:**
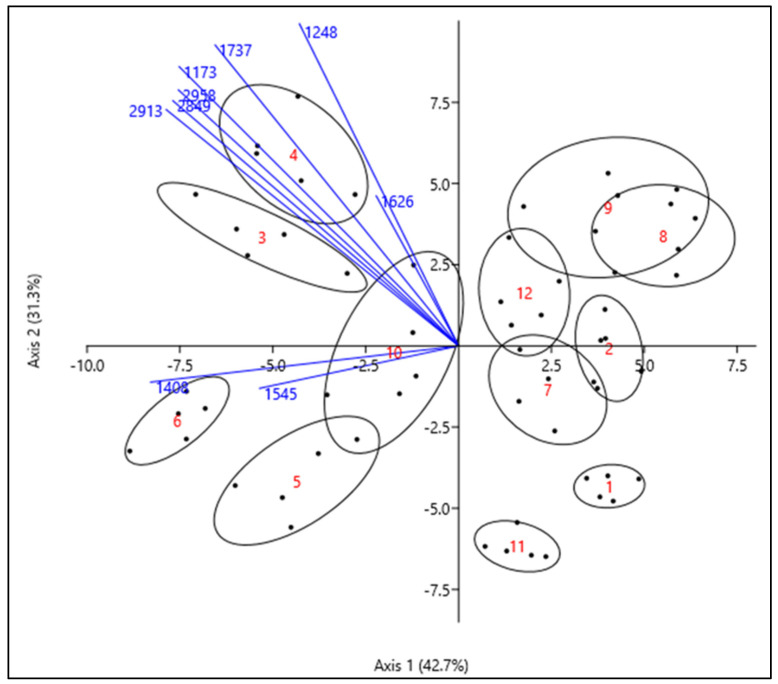
Discrimination analysis (LDA) based on the maximum (peak) absorbance values for the distinguished key ATR-FTIR ranges. The groups are marked with 60% ellipses. Wavenumbers (marked in blue) are explained in Table 1. Numbers from 1 to 12 denote the respective aphid species, in succession: 1—*Acyrthosiphon pisum*, 2—*Aphis fabae*, 3—*Aphis pomi*, 4—*Aphis viburni*, 5—*Aphis solanella,* 6—*Aphis spiraecola*, 7—*Brachycorynella asparagi*, 8—*Cinara cupressi*, 9—*Cinara tujafilina*, 10—*Macrosiphum rosae*, 11—*Macrosiphonella artemisiae*, 12—*Uroleucon obscurum*.

The LDA analysis showed that the best result for species differentiation of aphids is possible, based on a set of 9 key ATR-FTIR ranges (Table 1, Figure 4). It means that only one ATR-FTIR range established based on PCA loadings plots (1450–1480 cm^−1^, with a peak at 1471 cm^−1^) was useless.

LDA analysis of FTIR spectra of all species presented the differences in chemical composition between species (Figure 4). This analysis revealed a high efficiency of the method of distinguishing aphid species based on the ATR-FTIR spectra. The first two LDA axes covered 74% of the variance in the data. The first LDA axis (explained 42.7% of the variation) with high probability separated samples according to the 1408 cm^−1^ peak which corresponded mainly with lipids and, to a lesser extent, with 1545 cm^−1^ peak connected with amide II (mainly proteins). The second LDA axis explained 31.3 % of the variation and segregated the samples mainly according to the ATR-FTIR spectrum peak at 1737 cm^−1^ connected with lipids, peaks at 1173 and 1248 cm^−1^ corresponded with carbohydrates and the peak at 1626 cm^−1^ corresponded with amide I (Table 2, Figure 4).

Our research shows that the use of selected key ATR-FTIR ranges (peaks) allows for the correct classification of as many as 98% (with jackknifing procedure—90%) of the analyzed individuals. This means that in the methods we used, the distinguished key ATR-FTIR ranges are critical for the correct separation of aphid species based on the chemical structure of their body (Table S1).

## 4. Discussion

Spectrophotometric methods such as ATR-FTIR spectroscopy are often used in forensic entomology where unequivocal identification of specimens is an essential requirement. Many authors point to the possibility of quick identification of Diptera based on the unique spectra of biochemical composition, for example, for the identification and discrimination of Sarcophagidae species, where identification of larvae and adults is crucial for developing the monitoring and control of flesh fly species and in estimating the PMI [9]. Moreover, Warren et al. [28] used spectral measurements ranging from 400 to 1000 nm to identify changes within the developing larval stages of *Protophormia terraenovae* (Diptera, Calliphoridae), which can be used as a further reference for insect age estimation. Similar results were achieved when distinguishing larval stages of *Lucilla sericata* [29]. Furthermore, the correct classification of the species and life cycle stage of three fly species *Calliphora vomitoria, L. sericata* and *Musca domestica* and the correct identification of nine species of *Neodexiopsis* flies were demonstrated [22,30]. Studies using FTIR indicate that this method can be widely used to distinguish insects at different levels: genus, species, population or sex, but most were focused on flies or beetles. Our research used FTIR spectroscopy for the first time to differentiate aphid species. We correctly distinguished 12 species of aphid and found the method to be useful and efficient.

Earlier studies have shown that the spectroscopic method is very efficient and can distinguish individual species with over 80% accuracy depending on the order of the insects [23]. The author also points out that differences in morphological features, which have, to date, been the basis for distinguishing species, may be confirmed by differences resulting from the chemical composition of the insect [23]. Identification of most of the species performed to date with the use of spectrophotometry has made it possible to distinguish up to 11 species [11,23]. Within Hemiptera, specimens of seven species of leafhoppers were identified with 91.3% accuracy [24]. The application of nine selected regions allowed for 98% discrimination of the 12 tested aphid species (Figure 4). Such a high degree of efficiency in distinguishing different species is particularly important in the case of closely related species belonging to the same genus and often morphologically similar to each other, for example, species belonging to the genus *Aphis* sp. have all been correctly distinguished (Figure 4).

Our results showed differences in the chemical composition of the aphid body. Using FTIR spectroscopy, we showed the differences in the composition that allow for species separation. We have shown that molecular vibrations, which strongly correlated with LDA 1 and LDA 2, and corresponded with carbohydrates, lipids and amides, are the main factors that enable aphid species separation (Figure 4, Table 2). The carbohydrate-specific peaks (1173 and 1248 cm^−1^) correspond to chitin, which is one of the main components of the insect cuticle. Chitin is a glucose polysaccharide that, together with sclerotized proteins and wax, acts as scaffold material for the cuticle, creates the exoskeleton of insects and also supports the trachea and gut. The chemical composition of the cuticle may be a distinguishing feature of insect species, but it also changes with age, which, for example, was found in the species and age determination of mosquitos [3]. The analysis for the selected key ATR-FTIR region showed that the most useful range for aphid discrimination was also the lipid-specific band peaks (around 2958, 2913, 2849, 1737 and 1408 cm^−1^) (Figure 4, Table 1). The lipid components in the insect’s body play important roles, for example, as a backup energy source, most often in the form of triglycerides [31,32]. Fatty acids stored in the insect’s body fat can also play a role in the processes of diapause and overwintering [25]. Lipids also act as body surface protection lipids (cuticular lipids), which range from protection against the environment, such as control of water transpiration, to reduction of abrasive damage and prevention of pathogen, and also serve as a communication channel between the insects [33,34]. Generally, waxy secretions on the integument of aphids are thought to limit their contact with the sticky, sugary honeydew excreted from the same, or other, individuals in the colony, and possibly protect against fungi, parasitoids, predators, dehydration and frost [35,36]. Using ATR-FTIR spectroscopy, we have validated the importance of wax esters for species separation (peak 1737 cm^−1^). Species of aphids produce significant amounts of wax secretions to protect their bodies from honeydew contamination. The results showed that the peak 1737 cm^−1^, due to molecular vibrations of the carbonyl groups present, e.g., in esters, has a high degree of correlation with the LDA second axis, which indicates its great utility in the separation of aphid species (Table 2). The presence of molecular vibrations in this region was also observed in the woolly oak aphid *Stegophylla brevirostris* and bee’s wax [36]. It can be assumed that the differences in the degree of waxy secretions observed in different aphid species could be related to different mechanisms of protection against honeydew. Many species remove honeydew using morphological adaptations, which include removing droplets from the anus with the legs and shooting excreta from the anus, assisted by well-developed cauda [37]. Species that do not reject honeydew exhibit behavioral adaptations by interacting with ants that eat the honeydew and clean the aphid bodies [38]. The adhesive function of waxes protecting them from their honeydew is particularly visible in gall-inhabiting aphids. The characteristic absorption from cuticle lipids was helpful in classifying 11 species of beetles [11] and rapid discrimination of maggots [22]. The authors suggested that the separation between maggot species was mainly down to the stretch at 1741 cm^−1^ and the amide I at 1637 cm^−1^ and amide II at 1513 cm^−1^ [22]. The peak corresponding to amide II (1545 cm^−1^), which was correlated with the LDA first axis, was very useful in distinguishing species of aphids (Figure 4, Table 2). Earlier studies have shown that amide II was also very useful for distinguishing the *Ectatomma vizottoi* ant castes [14].

Our research indicates that this method could be used to discriminate against aphid species, but more research is needed. Many studies show that the chemical composition of the insect’s body changes during its life cycle, depending on gender, and adapts to unfavorable environmental conditions, e.g., the season [32,39,40]. Therefore, as in the case of plant material, it is important to properly collect and prepare samples for FTIR analysis, taking into account all these factors [41].

## 5. Conclusions

It has been shown that the use of FTIR spectroscopy enables the identification of aphid species, based on the differences in the chemical composition of aphid bodies. The main factors enabling the differentiation of the species of aphids are lipid-specific vibrations (peaks around 2958, 2913, 2849, 1737 and 1408 cm^−1^), amides I and II molecular vibrations (peaks around 1626 and 1545 cm^−1^) and molecular vibrations in carbohydrates (peaks around 1248 and 1173 cm^−1^). Nine absorption peaks have been established to enable the correct identification of the 12 aphid species.

## Figures and Tables

**Table 1:** Key ATR-FTIR ranges and peaks distinguished by PCA and LDA analysis. Peak assignments based on [3,5,25].

ATR-FTIR Range (cm^−1^)	Peak at Corresponding Wavenumber (cm^−1^)	Proposed Definition of the Spectral Assignments
2950–2960	2958	C-H stretching, asymmetric vibrations: (CH_3_) mainly from lipids
2910–2930	2913	C-H stretching, asymmetric vibrations: (CH_2_) mainly from lipids
2835–2860	2849	C-H stretching, symmetric vibrations: (CH_2_) mainly from lipids
1720–1750	1737	C=O stretching, symmetric vibrations: mainly from lipids
1620–1640	1626	C=O stretching vibrations of amide I: mainly from proteins
1500–1560	1545	C=N stretching vibrations of amide II and N-H bending vibrations: mainly from proteins
1390–1420	1408	C-H bending, asymmetric vibrations: (CH_3_) mainly from lipids
1230–1260	1248	O-H and C-H bending vibrations: mainly from carbohydrates
1145–1180	1173	C-O stretching vibrations: (C-OH, C-O-C) mainly from carbohydrates

**Table 2:** Correlation between LDA scores for the first two LDA axes and absorption peaks for distinguished key ATR-FTIR ranges using Spearman rank correlation test.

ATR-FTIR Peak at Corresponding Wavenumber (cm^−1^)	2958	2913	2849	1737	1626	1545	1408	1248	1173
LDA 1	−0.58 ***	−0.58 ***	−0.56 ***	−0.47 ***	−0.12	−0.43 ***	−0.69 ***	−0.31 *	−0.52 ***
LDA 2	0.55 ***	0.50 ***	0.52 ***	0.64 ***	0.32 *	−0.09	−0.08	0.72 ***	0.64 ***

* *p* ≤ 0.05, *** *p* ≤ 0.001.

## Data Availability

The data presented in this study are available in the article. Additional data are available on request from the corresponding author.

## Supplementary Materials

The following supporting information can be downloaded at: https://www.mdpi.com/article/10.3390/biology11081232/s1, Table S1. Results of aphid species classification based on LDA analysis. A and B—classification matrix obtained without and using jackknifing procedure. Rows: given groups; columns: predicted groups.
