# Supplementary material for: Highly Efficient Use of Infrared Spectroscopy (ATR-FTIR) to Identify Aphid Species
Source: Biology (Basel). 2022 Aug 18;11(8):1232. doi: 10.3390/biology11081232 (PMC9404783; doi:10.3390/biology11081232)
Supplement: Supplementary file 1 [file biology-11-01232-s001.zip › biology-1810948-supplementary.pdf]

Table S1. Results of aphid species classification based on LDA analysis. A and B - classification matrix obtained without and using jackknifing procedure. Rows: given groups; columns: predicted groups.

**A**	**1**	**2**	**3**	**4**	**5**	**6**	**7**	**8**	**9**	**10**	**12**	**11**	**Total**
**1**	5	0	0	0	0	0	0	0	0	0	0	0	5
**2**	0	5	0	0	0	0	0	0	0	0	0	0	5
**3**	0	0	5	0	0	0	0	0	0	0	0	0	5
**4**	0	0	0	5	0	0	0	0	0	0	0	0	5
**5**	0	0	0	0	5	0	0	0	0	0	0	0	5
**6**	0	0	0	0	0	5	0	0	0	0	0	0	5
**7**	0	0	0	0	0	0	5	0	0	0	0	0	5
**8**	0	0	0	0	0	0	0	4	1	0	0	0	5
**9**	0	0	0	0	0	0	0	0	5	0	0	0	5
**10**	0	0	0	0	0	0	0	0	0	5	0	0	5
**12**	0	0	0	0	0	0	0	0	0	0	5	0	5
**11**	0	0	0	0	0	0	0	0	0	0	0	5	5
Total	5	5	5	5	5	5	5	4	6	5	5	5	60

% correctly classified: 98.3

**B**	**1**	**2**	**3**	**4**	**5**	**6**	**7**	**8**	**9**	**10**	**12**	**11**	**Total**
**1**	5	0	0	0	0	0	0	0	0	0	0	0	5
**2**	0	5	0	0	0	0	0	0	0	0	0	0	5
**3**	0	0	4	1	0	0	0	0	0	0	0	0	5
**4**	0	0	0	5	0	0	0	0	0	0	0	0	5
**5**	0	0	0	0	5	0	0	0	0	0	0	0	5
**6**	0	0	0	0	0	5	0	0	0	0	0	0	5
**7**	1	0	0	0	0	0	4	0	0	0	0	0	5
**8**	0	0	0	0	0	0	0	4	1	0	0	0	5
**9**	0	0	0	0	0	0	0	1	3	0	1	0	5
**10**	0	0	0	0	0	0	0	0	0	4	1	0	5
**12**	0	0	0	0	0	0	0	0	0	0	5	0	5
**11**	0	0	0	0	0	0	0	0	0	0	0	5	5
Total	6	5	4	6	5	5	4	5	4	4	7	5	60

% correctly classified: 90.0
